# Involvement of the Androgen and Glucocorticoid Receptors in Bladder Cancer

**DOI:** 10.1155/2015/384860

**Published:** 2015-08-10

**Authors:** Lucien McBeth, Maria Grabnar, Steven Selman, Terry D. Hinds

**Affiliations:** ^1^Center for Hypertension and Personalized Medicine, Department of Physiology & Pharmacology, University of Toledo College of Medicine, Toledo, OH 43614, USA; ^2^Department of Urology, University of Toledo College of Medicine, Toledo, OH 43614, USA

## Abstract

Bladder cancer is encountered worldwide having been associated with a host of environmental and lifestyle risk factors. The disease has a male to female prevalence of 3 : 1. This disparity has raised the possibility of the androgen receptor (AR) pathway being involved in the genesis of the disease; indeed, research has shown that AR is involved in and is likely a driver of bladder cancer. Similarly, an inflammatory response has been implicated as a major player in bladder carcinogenesis. Consistent with this concept, recent work on anti-inflammatory glucocorticoid signaling points to a pathway that may impact bladder cancer. The glucocorticoid receptor- (GR-) *α* isoform has an important role in suppressing inflammatory processes, which may be attenuated by AR in the development of bladder cancer. In addition, a GR isoform that is inhibitory to GR*α*, GR*β*, is proinflammatory and has been shown to induce cancer growth. In this paper, we review the evidence of inflammatory mediators and the relationship of AR and GR isoforms as they relate to the propensity for bladder cancer.

## 1. Introduction

Bladder cancer is the sixth most common cancer in the United States [1]. It was predicted that there would be approximately 75,000 new cases and 16,000 deaths in 2014. There is a recognized predilection for males with an incidence ratio of 3 : 1. Urothelial carcinoma, arising in the mucosa of the bladder, accounts for the majority of cases encountered in the United States. There are three clinicopathologic forms of urothelial carcinomas: papillary, solid/nodular, and carcinoma in situ (CIS). Grading of these cancers is currently defined as either low or high grade depending upon standard histological findings. Patients with low-grade tumors generally have a favorable prognosis; however, the risk of recurrence is high (60–80%) [2]. High-grade tumors are much more aggressive with a proclivity for invasion and metastasis [3]. CIS by definition is high grade and its presence in combination with papillary or solid cancers can alter treatment paradigms and prognosis. Pathological staging is based on depth of bladder wall invasion. High grade and bladder wall muscle invasion are associated with poorer outcomes [4].

Recent investigations have shown that inflammation and proinflammatory cytokine production are correlated with advanced cases of cancer and may be indicators of a poor prognosis [5]. Proinflammatory cytokines, such as tumor necrosis factor alpha (TNF*α*) and interleukin-6 (IL-6), lead to an inflammatory state that stimulates tumor growth [6]. There is evidence that an isoform of the glucocorticoid receptor (GR), GR*β*, which is inhibitory to glucocorticoid action, increased by inflammation and may lead to cancer growth [7, 8]. Further studies have shown that GR*β* may also enhance androgen receptor (AR) induced growth in prostate cancer cells [8]. However, the relationship of AR and GR*β* has not been established. The importance of anti-inflammatory glucocorticoids in the management of bladder cancer is only now becoming understood. Herein, we will discuss the roles of the androgen and glucocorticoid nuclear receptor signaling pathways as they relate to inflammation and bladder cancer.

## 2. Factors Leading to Bladder Cancer

Inflammatory pathways and agents that cause inflammation are associated with bladder cancer, which include certain types of infections, environmental/lifestyle factors, and iatrogenic factors.

Inflammation from urinary tract infections caused by* Schistosoma haematobium*, which is a digenetic trematode found in Africa and the Middle East,is associated with a separate type of bladder cancer, squamous cell bladder cancer (also known as bilharzial bladder cancer) [9]. Heavy egg deposits in the bladder mucosa and submucosa occur during the acute phase of* S. haematobium* infection in humans [9, 10]. The eggs act as a mechanical irritant to the bladder epithelium, inducing chronic inflammatory lesions thus priming the bladder for inflammation and carcinogenesis [11]. There is a 5.6 : 1 male prevalence for the egg-induced* Schistosoma* bladder cancers (4.3 : 1 incidence in males for nonegg* Schistosoma*), which is greater than any other bladder squamous cell carcinoma in patients from Egypt [12]. This may be due to a higher male susceptibility, or more exposure of males to causative agents. Interestingly, the loss of the Y chromosome was observed in 7 of 17 (41%) male cases studied with* S. haematobium *induced bilharzial bladder cancer [13], indicating a unique male pathway in squamous cell bladder cancer development. However, there have been no investigations in patients with* S. haematobium *infection on the involvement of the androgen or glucocorticoid receptors.

Both lifestyle factors and environmental agents have been causally related to the development of bladder cancer. Interestingly, there is a male propensity in bladder cancer caused by cigarette smoking, which is estimated to contribute to 50% of cases in men and 35% in women [14]. Regular cystitis is positively associated with bladder cancer risk and may be due to chronic inflammation from carcinogens in the urine of patients that smoke [15]. Environmental or occupational exposure to various chemical carcinogens, such as aromatic amines and polycyclic aromatic hydrocarbons used in the production of aluminum, coal gasification, roofing, and carbon black manufacturing, is one of the agents known to potentially induce bladder cancer [16, 17], which is most likely through induction of inflammation and chronic cystitis in bladder. There is a separation in the amount of occupational chemical exposure in men versus women [18], which may indicate a predisposition of bladder cancer in males. However, the effect of chemical carcinogens on AR signaling activity in bladder is unknown.

Iatrogenic factors that cause bladder cancer include chemotherapeutic agents and radiation. Cyclophosphamide is widely used in a variety of clinical scenarios, which can form metabolites that can contribute to the development of bladder cancer [19]. The inactive metabolite of cyclophosphamide, acrolein, is excreted into urine which induces inflammation of the bladder leading to hemorrhagic cystitis [20]. Bladder epithelial damage occurs because of a reduction of endogenous glutathione and generation of free radicals that initiate lipid peroxidation and other cell damage. There have been no differences found in the treatment of cyclophosphamide and bladder cancer between males and females [21, 22]. In addition, treatment of rats with cyclophosphamide in males showed no significant difference with respect to male reproductive organ weights, serum testosterone, luteinizing hormone or follicle-stimulating hormone, epididymal sperm counts, or fertility [22]. However, cyclophosphamide has been shown to penetrate the male reproductive tract and can be transmitted sexually to a female partner, which may affect progeny outcome [21]. Other iatrogenic factors, such as chronic low-dose radiation, may also lead to bladder cancer through oxidative stress and a reduction in DNA repair by an increase of nitric oxide and reactive oxygen species [23–25]. Therapeutic pelvic radiation used for abnormal uterine bleeding and ovarian, cervical, and prostate cancer is associated with an increase in bladder cancer risk [26]. Altogether, iatrogenic factors insult the bladder, causing inflammation, resulting in DNA damage and mucosal aberrations leading to bladder cancer. However, there is no correlation for sexual prevalence that has been observed.

## 3. Current Therapies

Treatment of localized bladder cancer can vary from simple fulguration to multimodal therapy including radical extirpative therapy. A number of treatment paradigms exist depending on the clinical situation. Low-grade papillary tumors are handled frequently with simple electrodessication. Inflammation has been shown to play a role in bladder cancer and therapies that are immunomodulators have proven useful in treatment. Adjuvant intravesical therapy with either chemotherapeutic agents such as Mitomycin C or a biologic such as Bacillus Calmette Guerin (BCG) may be employed to prevent recurrences [27]. BCG has proved useful in the management of CIS and superficial high-grade papillary (noninvasive) cancers. BCG is commonly used to prevent bladder cancer recurrence after transurethral resection of the bladder tumor [2, 28, 29]. For high-grade lesions, treatment is based on the depth of invasion. Muscle invasion usually leads to a much more radical treatment including neoadjuvant chemotherapy combined with surgical removal of the bladder or radiation therapy.

## 4. Inflammatory Pathways and Bladder Cancer

BCG is a weakened vaccine strain of bovine tuberculosis from* Mycobacterium bovis* that functions as an immunotherapy to redirect the immune system to clear bladder cancer cells (reviewed in [30]). It has been shown that internalization of BCG by urothelial cells enhanced the expression of the major histocompatibility complex (MHC) class II and cluster of differentiation 1 (CD1) proteins [31], thus, indicating that endothelial cells can present more antigens of BCG infection and likely tumor presence. However, up to 40% of patients fail to respond to immunotherapy [32]. In males, BCG treatment has been associated with relatively rare complications of penile edema and meatal ulceration [33], as well as epididymoorchitis [34, 35]. BCG treatment has been shown to be detrimental to healthy sperm development in young men following therapy [36]. In addition, intratesticular injection of BCG in dogs caused a severe granulomatous reaction with widespread degeneration of the tubules, resulting in azoospermia [37]. However, the response to BCG treatment in men and women has shown similar results.

To prevent tumors, macrophages must migrate in the area surrounding the tumor [38]. The exact role that macrophages play depends on which subtype they belong to, as there are pro- and anti-inflammatory types [38] (reviewed in [39, 40]). Insults that induce bladder inflammation without host-derived secreted protein acidic and rich in cysteine (SPARC) cause activation of proinflammatory macrophages and nuclear factor kappa-light-chain-enhancer of activated B cells (NF-*κ*B) [41, 42], which enhances growth of bladder cells. However, proinflammatory macrophages can also regulate cancer growth. Type 1 (proinflammatory) macrophages cocultured with human bladder cancer cells arrested cancer cell growth and increased TNF*α* expression and phosphoinositide 3-kinase (PI3K)/protein kinase B (Akt) signaling pathway activity when compared to cancer cells grown alone and cancer cells cocultured with type 2 (anti-inflammatory) macrophages [43].

**Figure 1:**
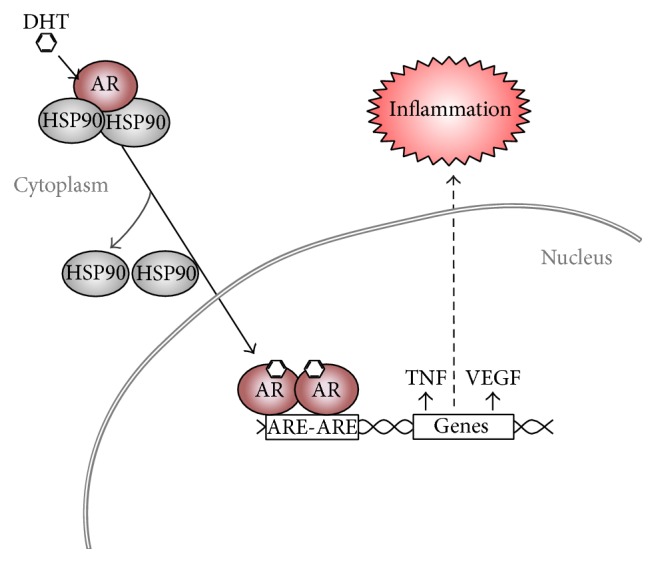
Activation of the androgen receptor leads to inflammation. Activation by androgens causes translocation of AR from the cytoplasm to the nucleus and release of HSP90 chaperone proteins. The AR then binds to androgen-response elements (ARE) in the promoter region of genes resulting in an increase or suppression. TNF*α* and VEGF are two genes that contain AREs in their promoter and are activated by AR to increase inflammatory signals.

AR can be proinflammatory by inducing expression of genes such as TNF*α* [44], which can enhance immune cell invasion and may contribute to chronic inflammation (Figure 1). Several proteins, including the AR which is found in both bladder stromal cells and the urothelium, are known to contribute to bladder cancer growth [45, 46]. The effect of GR*β* on AR guided proinflammatory pathways in bladder cancer remains unknown. However, Ligr et al. recently showed that GR*β* can increase AR regulated growth in prostate cancer cells [8]. Future studies on the relationship of GR*β* and AR would strengthen our understanding if they work in conjunction to inhibit the anti-inflammatory actions of glucocorticoids.

**Figure 2:**
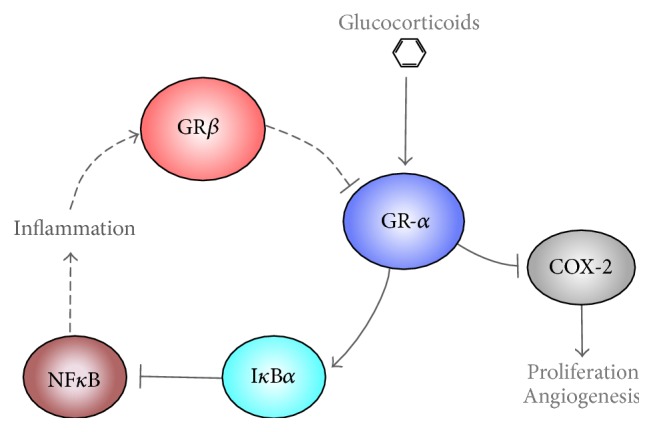
Glucocorticoids suppress inflammation. Glucocorticoids bind and activate GR*α*, which enhances I*κ*B*α* and suppresses COX-2 to inhibit inflammation. I*κ*B*α* binds to inhibit NF*κ*B, the major mediator of inflammation. NF*κ*B increases GR*β* to cause glucocorticoid resistance and proliferation.

The use of nonsteroidal anti-inflammatory drugs is inversely associated with bladder cancer due to their inhibition of the cyclooxygenase-2 (COX-2) inflammatory pathway [47]. Overexpression of COX-2 is associated with proliferation, angiogenesis, and dysregulation of apoptosis in bladder cancer cells and is upregulated in bladder epithelial cancer [48–50]. Interferon-*α* (IFN-*α*) decreased expression of COX-1 and increased COX-2 in bladder cancer cells, suggesting that IFN-*α* plays a role in COX-2 upregulation in urothelial cancer cells [50]. Glucocorticoids are inhibitors of COX-2 expression (Figure 2) [51], suggesting that they may be useful for inhibition of bladder cancer. Glucocorticoids also have a beneficial anti-inflammatory response by increasing I*κ*B*α*, an inhibitor of proinflammatory NF-*κ*B [52].

## 5. Glucocorticoids and Bladder Cancer

Glucocorticoids are commonly used drugs for treatment of inflammatory and autoimmune disorders. GR is expressed as two alternate major isoforms, GR*α* and GR*β* [53–56]. Glucocorticoids control anti-inflammatory cellular processes by binding to and activating GR*α.* Antiproliferative properties of glucocorticoids are mediated through GR*α*, which is a hormone-activated transcription factor [57, 58] that increases cell cycle arrest proteins p27 and p21 [59, 60] as well as the apoptotic-gene phosphatase and tensin homolog deleted on chromosome 10 (PTEN) [61]. In contrast to GR*α*, GR*β* lacks part of the ligand-binding domain, helix 12, of the GR protein and cannot bind glucocorticoids [55]. Although the function of GR*β* is not well understood, it has been shown that GR*β* acts as an inhibitor to GR*α* [55, 56, 62–64]. GR*β* is induced by inflammatory pathways such as TNF*α* and NF-*κ*B [7], suggesting that it may have a paramount role in inflammation that is associated with bladder cancer (Figure 2). The inhibitory role of GR*β* on glucocorticoid action in the immune system has related it to a variety of immunological diseases, such as ulcerative colitis, asthma, and chronic sinusitis [54, 65–68]. Now, it is also being observed that GR*β* may regulate proliferation as well as cancer growth in glioblastoma [65] and leukemia [67]. Potentially, this may occur through the ability of GR*β* to augment a chronic inflammatory state by inhibition of GR*α* and glucocorticoid action.

**Figure 3:**
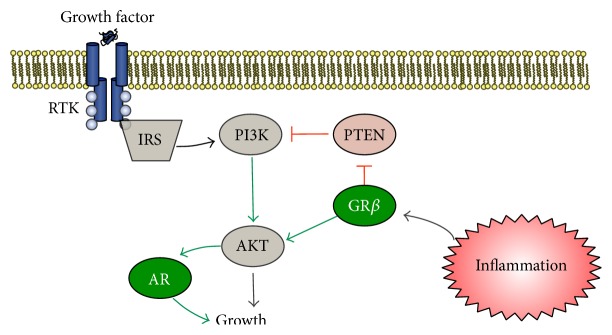
Pathway in males that leads to growth factor activation of PI3K/AKT of AR and GR*β* induced growth. Growth factors (e.g., insulin, epidermal growth factor) bind to receptor tyrosine kinases (RTK) increasing phosphorylation and activation of insulin receptor substrate (IRS), resulting in the induction of the PI3Kinase growth pathway. PTEN is a tumor suppressor gene that inhibits PI3K. The PI3K increases activity of AKT resulting in enhanced AR signaling and growth. Proinflammatory mediators increase expression of GR*β*, which has been shown to inhibit the tumor suppressor gene, PTEN [122], which leads to enhanced AR induced growth [56].

It has been shown that GR plays a role in bladder cancer. However, the precise mechanism and isoform of the receptor is responsible is unknown. GR expression tends to be weaker in bladder cancer tumors than in normal cells, and strong GR expression tends to be correlated with a better prognosis [69, 70]. Glucocorticoids have been widely used as comedication in patients with advanced bladder cancer [71]. However, recent studies have raised the possibility of an increased risk of bladder cancer from systemic use of glucocorticoids. Recent investigations have demonstrated that glucocorticoids (e.g., corticosterone, dexamethasone, and prednisone) suppress bladder cancer cell invasion, while dexamethasone may induce proliferation via inhibiting apoptosis [72, 73]. However, these effects may be mediated by GR*β*, which has been shown to exert a stimulatory effect on proliferation [55], possibly by increasing inflammation in bladder by inhibition of GR*α*. Glucocorticoids are known to interfere with the transcriptional activity of several immune related transcription factors, including NF-*κ*B [69]. It has been shown that GR*α* can directly function as a corepressor of NF-*κ*B. Additionally, the synthetic glucocorticoid dexamethasone inactivates NF-*κ*B and downregulates NF-*κ*B-dependent cytokine IL-6, which may be a central mechanism involved in GR-mediated inhibition of bladder cancer cell invasion [69]. We have shown that dexamethasone can increase the expression of GR*β* in mouse embryonic fibroblast cells [55]. Interestingly, constant exposure of glucocorticoids in patients leads to elevated GR*β* and glucocorticoid resistance, which is due to decreased affinity for GCs and increased total GR proteins [62, 74], suggesting a chronic glucocorticoid resistant state. Furthermore, GR*β* can regulate growth through suppression of PTEN, enhancing PI3Kinase/AKT induced proliferation [56]. Suppression of GR*β* by siRNA inhibited growth of AR positive prostate cancer cells [8]. This suggests that GR*β* may positively affect AR signaling activity and that chronic glucocorticoid treatment in males could result in activation of the GR*β*/AR axis leading to bladder cancer (Figure 3).

Several glucocorticoids have been used clinically as cytotoxic agents, predominantly for hematologic malignancies [75]. Evidence suggests a glucocorticoid-induced resistance to cytotoxic effects of the antineoplastic drug* cis*-diamminedichloroplatinum (CDDP), the most effective agent currently used against urothelial carcinoma [76]. A glucocorticoid is often used as comedication in the standard chemotherapy regimens for bladder cancer, due to its protective factor against toxic chemotherapy drugs. However, prolonged systemic use of glucocorticoids has been shown to increase the subsequent risk of bladder cancer, possibly due to immunosuppression [71] or long-term induction of GR*β* causing glucocorticoid resistance leading to inflammation. However, the exact mechanism remains unknown.

## 6. Androgens and Bladder Cancer

In males, the AR has been shown to play a key role in prostate cancer genesis and progression [77]. However, the role of AR in bladder cancer and the proclivity for males has only recently drawn attention [78, 79]. AR is a ligand-inducible transcription factor that regulates the expression of several genes (Figure 1) [80–82]. AR ligands, the principal being the predominately male hormone, testosterone, enter the target cell and bind to AR directly or after conversion to 5*α*-dihydrotestosterone (DHT). The ligand-AR complex induces a conformational change in AR, resulting in release of the heat shock proteins (HSPs) and translocation of the complex from the cytoplasm to the nucleus [83]. After translocation, activated AR binds to DNA at androgen-response elements in promoters and recruits additional proteins, leading to specific transcriptional activation or repression of target genes [84]. Several human bladder cancer cell lines have been found to express AR [85–89]. Additionally, AR expression has been detected in human bladder cancer obtained after surgical removal [90–94]. The role of AR in normal bladder is unclear [95].

The role of AR in prostate has been more defined. Investigations have shown that ligand-independent activation of the AR pathway occurs in prostate cancer, which can be enhanced by epidermal growth factor (EGF) through the signal transduction pathway [81]. Connecting AR in prostate and bladder cancers, dysregulation of the epidermal growth factor receptor (EGFR) is associated with bladder cancer [96], suggesting that these cancer pathways may be interconnected. It has been shown that AR can increase expression and activity of EGFR and a protein encoded by the ERBB2 (also known as Her2) gene [96], implying androgen-mediated bladder cancer tumorigenesis and clinical progression via the regulation of the EGFR/ERBB2 pathways. The separation of these pathways in males and females is unknown. However, it may be overly activated in males because of the increased level of androgens.

Earlier investigators have shown that a variety of AR gene alterations are important in the development of bladder cancer, such as allelic loss and gene mutation. This could explain some of the differences between male and female tumors. Allelic loss of the AR locus has been found in cases of muscle-invasive bladder tumors, but not in the adjacent nonneoplastic tissue [97]. Additionally, mRNAs from two human bladder cancer cell lines have revealed AR sequences with short CAG repeat lengths, suggesting that altered mRNA sequences of the AR gene could contribute to bladder cancer [88]. Demonstrating the susceptibility of males, bladder cancer tumors implanted in rats that were treated with androgenic hormones grew more rapidly than rats treated with estrogenic hormones [98]. This was also supported by two studies in mice using AR knockout animals, which indicated a critical role of androgen signaling in bladder carcinogenesis [86, 99]. It is therefore suggested that androgenic hormones stimulate bladder tumor growth, whereas estrogenic hormones may do the opposite (or at least do not stimulate). However, there is evidence of AR induced bladder cancer in females. A study of transitional cell carcinoma showed that AR is expressed in women patients and found that 30% of the bladder cancer tumors are AR positive and that nontumor tissue may also express AR [100]. In addition, the same relationship between AR level and pathological stage was found in men and women.

The estrogen receptor (ER) *β* has been shown to be highly expressed in bladder cancer, with elevated ER*β* expression being correlated with increased bladder cancer stage [101]. In addition, it has been shown that ER*β* selective antiestrogen drug, raloxifene, causes bladder cancer cells to undergo apoptosis [102]. This shows that the use of antiestrogen therapy may be useful in treating bladder cancer; however, ER*β* has not been shown to be a driver of bladder cancer, while AR's driving capability has been demonstrated. ER*α* has been shown to interact with GR in breast, where this isoform is dominant [103]. However, no work has been done showing ER*β* and GR interaction.

Targeting of AR may potentially be a good therapy for bladder cancer in males. An effective prostate cancer treatment is chemical castration using luteinizing hormone-releasing hormone analogues to ablate testicular androgens or use of antiandrogens (e.g., flutamide), which block androgen signaling at the level of the AR. Typically, antiandrogens are used in early stage prostate cancer. While this therapy is successful at first, the hormonal therapy often fails and patients relapse with “castrate-resistant” prostate cancer. This resistance comes from the selection of cells that bypass androgen requirement by mechanisms including AR gene mutation or receptor amplification [104–108]. Additionally, it has been found that dihydrotestosterone upregulates ERBB2 in androgen receptor positive bladder cancer cells [109]. The communication between the AR and EGFR pathways may play a role in the male prevalence in bladder cancer. While it is known that AR positively correlates with an increased risk of developing prostate cancer, it is unknown whether antiandrogens can have an effect on bladder cancer.

## 7. Sexual Dimorphism in Cancer Aggressiveness

Males have been shown to develop more high-grade bladder cancer tumors in comparison to females (55.7% males versus 42.0% females) as well as a greater percentage of invasive tumors (26.5% males versus 22.0% females) [96]. Recent research has shown that androgens and AR can induce epithelial-mesenchymal transition (EMT) which is often seen as an indicator for metastasis [110, 111]. The aggressiveness of tumors is derived from oxygen and other nutrients that cancer cells use to induce local neovascularization. Vascular endothelial growth factor (VEGF) is a potent endothelial cell mitogen that stimulates proliferation, migration, and tube formation leading to angiogenic growth of new blood vessels and is essential during development [112]. Neuropilin-1 (NRP-1) and homologue NRP-2 are coreceptors that enhance responses to several growth factors, such as VEGF, and mediators under physiological and pathological conditions [113–116]. NRPs and VEGF receptors are constituently expressed on normal bladder epithelial cells and have been shown to be upregulated in an animal model of chronically inflamed cells, indicating neovascularization [117]. NRPs can regulate the cancer-induced vascular and inflammatory responses. Glucocorticoids have been shown to suppress NRP [118] and VEGF expression [73, 119]. Androgens, on the other hand, increase VEGF expression [120]. In males, GR*β* may enhance neovascularization potential through inhibition of GR*α*, as well as activation of AR and inflammatory pathways that increase VEGF and NRP. We have shown that GR*β* does enhance activity of the PI3-kinase and Akt cascade by suppression of PTEN (Figure 3) [121], a known inhibitor of growth and tumor suppressor gene [56]. Ultimately, this may lead to the inflammatory processes that can lead to the progression of bladder cancer in males.

## 8. Conclusions

Insight into the cellular biology of the bladder cancer disease process offers the opportunity to develop innovative and more targeted therapies. The androgen and the glucocorticoid receptors are both members of the steroid receptor superfamily and appear to offer promise as therapeutic targets for enhanced treatment paradigms. Much research remains to be performed in order to define the roles of glucocorticoids, antiandrogens, and the GR isoforms in the management of bladder cancer. A continuing understanding of the roles of AR and other molecules, such as GR*β*, that may directly or indirectly regulate androgens may help reveal better strategies for the management of bladder cancer in males. Additionally, androgen ablation therapy may prove to be useful for treatment in males with bladder cancer. A further understanding of the molecular signaling pathways that cause the predilection for males will aid in the advancement of bladder cancer therapy.
